# Bilateral approach selection in neuroendoscopic surgery for pituitary adenomas and health economic evaluation

**DOI:** 10.3389/fsurg.2026.1720182

**Published:** 2026-04-29

**Authors:** Mingjian Li, Jiahui Liu, Lianshu Ding, Jing Xu, Yanxia Deng, Pengcheng Wang

**Affiliations:** 1 Department of Neurosurgery, WeiFang People’s Hospital, Shandong Second Medical University, Weifang, Shandong, China; 2 Department of Neurosurgery, The Affiliated Huai'an NO.1 People’s Hospital of Nanjing Medical University, Huaian, Jiangsu, China

**Keywords:** bilateral transsphenoidal approach, health economic evaluation, medical burden, neuroendoscopy, pituitary adenoma

## Abstract

**Objective:**

To investigate the impact of bilateral approach selection in neuroendoscopic transsphenoidal surgery for pituitary adenomas on patient prognosis and to analyze the medical burden on patients from a health economic perspective.

**Methods:**

A retrospective analysis was conducted on the data of 197 patients who underwent pituitary adenoma surgery. The patients were divided into two groups based on the surgical approach: the transseptal approach group (*n* = 108) and the bilateral nostril expanded transsphenoidal approach group (*n* = 89). The medical burden, clinical efficacy, surgical indicators, hormone levels, and complications were compared between the two groups.

**Results:**

Compared with the bilateral nostril expanded transsphenoidal approach, the transseptal approach was associated with significantly less intraoperative blood loss and shorter operative time (*P* < 0.05). No significant differences were observed in total medical costs, psychological burden, hormone profiles, or complication rates. Postoperative nasal packing was associated with reduced rates of diabetes insipidus and thyroid-stimulating hormone abnormalities (*P* < 0.05) and a marginally significant reduction in cerebrospinal fluid rhinorrhea (*P* = 0.05).

**Conclusion:**

The transseptal approach in pituitary adenoma surgery has the advantages of less intraoperative bleeding and shorter surgical duration, which can reduce postoperative anxiety and depression in patients. Postoperative nasal packing may reduce complications, but larger multicenter studies are warranted. Pituitary adenoma patients bear substantial economic and psychological burdens; multidisciplinary collaboration and pharmacoeconomic optimization are needed to reduce overall costs and improve outcomes.

## 1 Introduction

Pituitary adenomas are the most common benign tumors in the sellar region, with an increasing incidence rate. Although pituitary adenomas are benign, 30%–45% of them are invasive (1). These tumors, due to their unique anatomical structure, often lead to tumor recurrence or hormonal abnormalities. With the advancement of medical technology, the treatment methods for pituitary adenomas have become more diverse. However, the medical burden faced by patients and the uneven distribution of treatment resources still need in-depth discussion (2).

Nowadays, the endonasal neuroendoscopic surgery for pituitary adenomas has been recognized by doctors at all levels due to its advantages of less surgical trauma and faster postoperative recovery (3). However, there is still controversy over the selection of different nasal instrument approaches and the effect of postoperative gauze packing. How to reduce postoperative complications remains a key issue to be solved (4).

Based on the above situation, we retrospectively analyzed the medical burden and treatment of pituitary adenoma surgery patients to better utilize medical and health resources and reduce social burden.

## 2 Materials and methods

### 2.1 General information

A retrospective analysis was conducted on 197 patients with pituitary adenomas who underwent neuroendoscopic transsphenoidal surgery at Huai'an First People's Hospital from January 1, 2020, to March 31, 2024. This single-center retrospective cohort study was not randomized. To mitigate selection bias, we performed propensity-score matching (PSM) with a caliper of 0.02, adjusting for age, sex, tumor size, and Knosp grade. Given the limited sample size in the bilateral approach group, we employed a variable ratio matching strategy (1:1 to 1:2) to optimize statistical power, resulting in 108 transseptal patients matched to 89 bilateral approach patients. After matching, covariate balance was assessed by standardized mean differences (<0.1 considered acceptable), and the difference in the primary outcome remained consistent with that observed in the full cohort. Sensitivity analyses using the full unmatched cohort with multivariable regression adjustment confirmed the robustness of our findings. Inclusion criteria: 1. Underwent neuroendoscopic transsphenoidal surgery; 2. No olfactory loss before surgery, and postoperative pathology indicated pituitary adenoma; 3. Complete data. Exclusion criteria: 1. Combined with other intracranial tumors; 2. Congenital anosmia or nasal inflammation; 3. Did not obtain complete follow-up or missing data. This study was approved by the Ethics Committee of Huai'an First People's Hospital (IRB approval number: KY-2025-049-01).

### 2.2 Methods

All surgeries were performed by the same surgeon. The selection of surgical approach was based on preoperative MRI findings, specifically Knosp grade and Hardy classification. The transseptal approach was preferentially employed for Knosp grade 0–2 adenomas with predominantly intrasellar or minimal suprasellar extension. The bilateral-nostril expanded transsphenoidal approach was selected for tumors requiring wider bilateral exposure, extensive parasellar extension, or when intraoperative conversion was anticipated. All surgical decisions were made by the senior surgeon with >10 years of experience in endoscopic skull base surgery. Preoperative, all patients underwent imaging, hormone testing, visual field examination, and psychological testing. Olfactory tests were conducted three days before surgery, and the nasal cavity was cleaned and disinfected. During surgery, patients were in a supine position under general anesthesia. Adrenaline physiological saline cotton strips were used to constrict the bilateral nasal cavity. The tumor cavity was filled with gelatin sponge, the sellar floor was repaired with artificial dura mater, and dura mater glue was sprayed externally. For bilateral nasal cavities, the surgeon chose whether to place iodine gauze strips or not. Postoperative follow-up was conducted for patients to investigate the medical burden of pituitary adenomas.

#### 2.2.1 Transseptal approach

The opening of the sphenoid sinus mucosa in the sphenoethmoid recess between the middle turbinate and the nasal septum was identified, and a 1.5–2.0 cm incision was made laterally. The left nasal cavity was entered, and the nasal septum bone and mucosa were fully separated. A window was made adjacent to the anterior wall, and the anterior wall of the sphenoid sinus was removed to enter the sphenoid sinus cavity.

#### 2.2.2 Bilateral nostril expanded transsphenoidal approach

Both nasal cavities were used as the surgical pathway. The nasal mucosa was incised 1 cm to the posterior of the right nasal vestibule in an arc shape, separated to the posterior of the anterior wall of the sphenoid bone, and the bony nasal septum was fractured. The mucosa on the opposite side was separated, and a window was made at the anterior wall of the sphenoid sinus on the left side, and the anterior wall and septum of the sphenoid sinus were removed with a grinding drill.

### 2.3 Observation indicators and evaluation criteria

1. **Medical Burden:** Direct medical costs were calculated in Chinese Yuan (CNY) for fiscal year 2023, including: (i) Hospitalization costs: total costs, drug costs (Western and traditional Chinese medicine), surgical fees, and disposable consumables; (ii) Outpatient follow-up costs: registration fees, pituitary MRI, full-panel hormone tests, and visual-field examinations at 1, 3, 6, and 12 months post-surgery. The follow-up period was uniformly set at 12 months to ensure comparability. Indirect costs (lost wages, transportation) were not included. Costs were not adjusted for inflation given the 4-year study period. Patient psychological burden was assessed using the Hospital Anxiety and Depression Scale (HADS) (5).
2. **Surgical Parameters:** Surgical approach, whether iodine gauze strips were placed, tumor resection rate, preoperative and postoperative hormone levels, visual field conditions, and olfactory tests [using the T&T standard olfactory function test method (6) to assess patients’ olfactory function 3 days before surgery, 3 days after surgery, 1 week after surgery, and 1 month after surgery].
3. **Postoperative Complications:** Postoperative diabetes insipidus, cerebrospinal fluid rhinorrhea, intracranial infection, and nasal bleeding were recorded.
4. Long-term economic burden: Due to the retrospective design and limited follow-up duration (12 months), comprehensive long-term cost assessment including productivity loss, quality-adjusted life years (QALYs), and lifetime hormone replacement costs could not be fully captured. We acknowledge this as a significant limitation. Costs are presented in Chinese Yuan (CNY) for fiscal year 2023; approximate US Dollar equivalents [1 USD ≈ 6.9 CNY, 2023 average] are provided in parentheses for international comparability: total medical cost ∼$5,168 ($4,261-$5,558), drug cost ∼$1,179 ($635-$1,565), surgical cost ∼$1,118 ($818-$1,377).

### 2.4 Statistical methods

All analyses were conducted with R version 4.3.2. Categorical variables are presented as counts (percentages) and compared between groups using the *χ*^2^ test or Fisher's exact test, as appropriate. Continuous variables are reported as median (interquartile range) and were compared with the independent-samples t test or the Mann–Whitney U test according to distribution. A two-sided *P* value < 0.05 was considered statistically significant.

## 3 Results

**Figure 1:**
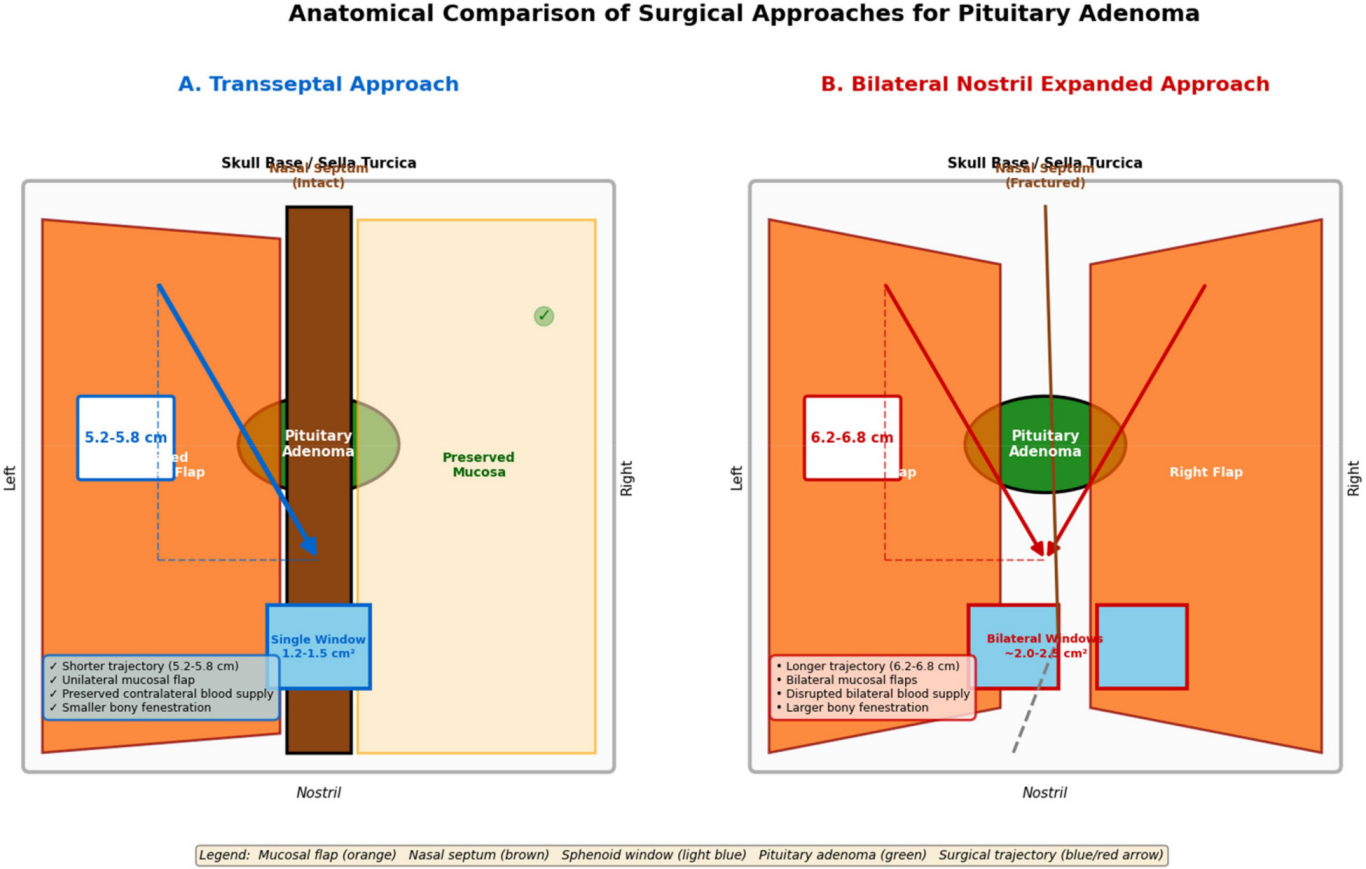
Anatomical comparison of surgical approaches for pituitary adenoma. Schematic illustration of the two endonasal transsphenoidal approaches. **(A)** The transseptal approach utilizes a unilateral mucosal flap with preservation of the contralateral nasal mucosa and sphenopalatine artery branches, resulting in a shorter working distance (5.2–5.8 cm) and unilateral sphenoidotomy (1.2–1.5 cm^2^). **(B)** The bilateral nostril expanded approach requires elevation of bilateral mucosal flaps, fracturing of the bony nasal septum, and creates a longer working distance (6.2–6.8 cm) with bilateral sphenoidotomy (∼2.0–2.5 cm^2^).

The anatomical differences between the transseptal and bilateral nostril expanded approaches (Figure 1) translate into distinct clinical outcomes. As detailed below, the shorter trajectory and unilateral dissection of the transseptal approach were associated with reduced operative time and blood loss, while both approaches demonstrated comparable efficacy in tumor resection and endocrine outcomes.

### 3.1 General information and medical burden of patients

The median age of all patients with pituitary adenomas was 57 years, with 104 (52.8%) female patients. The average hospitalization was 14 days, and the average total medical cost during hospitalization was 35,657 yuan, including drug costs (including traditional Chinese medicine and Western medicine) of 8,133 yuan, surgical treatment costs of 7,715 yuan, and surgical disposable consumable costs of 10,142 yuan. The total outpatient follow-up cost (including preoperative and postoperative follow-up costs) was 1,894 yuan. The median HADS score for all surgical patients before surgery was 8. No significant difference was observed between the two approaches (*P* = 0.56), suggesting that psychological burden is related to the disease and surgical experience overall rather than the specific nasal corridor selected. The general information and medical burden parameters of the two surgical approaches are shown in Table 1.

**Table 1:** General information and medical burden parameters of the Two surgical approaches.

Item	Transseptal (*n* = 108)	Bilateral (*n* = 89)	*P* value
Age (years)	55.2 ± 11.2	55.2 ± 11.0	0.97
Gender			0.89
Male	50 (46%)	43 (48%)	
Female	58 (54%)	46 (52%)	
Hospitalization Days	13.8 ± 10.8	13.2 ± 5.3	0.67
Total Medical Cost (CNY)	33,828 (29,316–38,345)	32,768 (28,401–37,190)	0.77
Drug Cost (CNY)	5,978 (4,379–8,945)	8,365 (5,760–10,795)	0.42
Surgical Treatment Cost (CNY)	7,715 (5,938–9,504)	6,736 (5,644–8,806)	0.74
Surgical Disposable Consumable Cost (CNY)	8,826 (6,219–12,464)	8,075 (6,056–11,937)	0.28
Outpatient Follow-up Total Cost (CNY)	1,799 (1,772–1,836)	1,800 (1,777–1,834)	0.55
HADS Score	8 (5–8)	8 (5–10)	0.56

Continuous variables are presented as mean ± standard deviation for normally distributed data (Shapiro–Wilk *P* > 0.05), or median (interquartile range) for non-normally distributed data. HADS scores and medical costs showed non-normal distribution and are presented as median (IQR). Outpatient follow-up cost refers to the sum of registration fees, pituitary MRI, full-panel pituitary-target-gland hormones, and visual-field tests at 1, 3, 6, and 12 months after surgery. The follow-up period was uniformly set at 12 months to ensure comparability. HADS (Hospital Anxiety and Depression Scale) uses the Zigmond & Snaith cut-off: 0–7 = no anxiety/depression; 8–10 = borderline; ≥11 = definite anxiety or depression. Scores ≥8 are considered indicative of mood disorder in this study.

### 3.2 Comparison of surgical data and complications between the two approaches

The median surgical time for the transseptal approach was 122 min, and the intraoperative blood loss was 39 mL. For the bilateral nostril expanded transsphenoidal approach, the median surgical time was 133 min, and the intraoperative blood loss was 59 mL. The differences were statistically significant. There were no statistically significant differences in tumor resection rate, postoperative complications, and olfactory tests. See Table 2. Comparison of hormone levels between the two approaches, see Table 3.

**Table 2:** Comparison of surgical data and complications between the Two approaches.

Item	Transseptal (*n* = 108)	Bilateral (*n* = 89)	t/*χ*^2^/z value	*P* value
Surgical Time (min)	117.5 ± 23.9	134.6 ± 21.1	−5.35	<0.01
Intraoperative Blood Loss (mL)	39.6 ± 9.7	59.9 ± 9.5	−14.80	<0.01
Tumor Resection Rate (%)	87.4 ± 6.7	86.9 ± 7.3	0.55	0.58
Diabetes Insipidus	64 (59%)	43 (48%)	2.07	0.16
Cerebrospinal Fluid Rhinorrhea	12 (11%)	15 (17%)	0.90	0.34
Intracranial Infection	1 (1%)	5 (6%)	2.16	0.14
Visual Field Damage	1 (1%)	0 (0%)	—	1.00
Olfactory Test (T&T Score)				
3 Days Postoperatively	1.30 (0.70–1.60)	1.00 (0.50–1.50)	1.26	0.21
1 Week Postoperatively	2.00 (1.60–2.60)	2.10 (1.60–2.60)	0.05	0.96
1 Month Postoperatively	3.30 (2.77–3.80)	3.10 (2.70–4.00)	0.21	0.83

Data are presented as mean ± standard deviation, median (interquartile range), or *n* (%). Surgical time, blood loss, and tumor resection rate showed normal distribution and are presented as mean ± SD. Olfactory T&T scores showed non-normal distribution and are presented as median (IQR). Categorical variables are compared using χ^2^ test or Fisher's exact test. Continuous variables are compared using independent-samples t test or Mann–Whitney U test according to distribution.

**Table 3:** Comparison of hormone levels between the two approaches.

Surgical Approach	Pre-operative hormone abnormalities (%)				Post-operative hormone abnormalities (%)			
	Prolactin	TSH	FSH	GH	Prolactin	TSH	FSH	GH
Transseptal (*n* = 108)	79 (73.1%)	4 (3.7%)	46 (42.6%)	45 (41.7%)	47 (43.5%)	6 (5.5%)	13 (12%)	6 (5.6%)
Bilateral nostril expanded (*n* = 89)	50 (56.2%)	5 (5.6%)	41 (46.1%)	39 (43.8%)	43 (48.3%)	14 (15.7%)	9 (10.1%)	2 (2.2%)
χ^2^ value	4.990	0.426	0.048	0.031	0.092	4.310	0.473	1.98
*P* value	0.03	0.51	0.83	0.86	0.76	0.04	0.49	0.29

Data are presented as *n* (%). TSH, Thyroid-Stimulating Hormone; FSH: follicle-stimulating hormone; GH, growth hormone.

While the 20-mL difference in intraoperative blood loss was statistically significant (*P* < 0.01), we acknowledge that this difference is clinically modest in isolation and unlikely to independently impact patient survival or major recovery outcomes. However, in the context of endoscopic skull base surgery, even modest blood reduction may improve visualization, reduce operative stress, and serve as a surrogate marker for surgical efficiency and tissue trauma. When combined with the 11-minute operative time reduction, these factors collectively suggest reduced procedural invasiveness with the transseptal approach.

To address potential confounding by tumor complexity, we performed subgroup analyses stratified by Knosp grade see Table 4. When stratified by Knosp grade (0–2 vs. 3–4), the transseptal approach demonstrated significantly shorter operative time and reduced blood loss in the Knosp 0–2 subgroup (*n* = 140; *P* < 0.01 for both). In Knosp 3−4 tumors, the difference in operative time remained significant (*P* = 0.03), but blood loss difference was attenuated (*P* = 0.08), suggesting reduced advantage in more complex cases. These findings support our recommendation for careful preoperative stratification.

**Table 4:** Subgroup analysis by knosp grade.

Parameter	Knosp Grade	Transseptal Approach	Bilateral Nostril Expanded Approach	t value	*P* value
Operative time (min)	0–2 (*n* = 140)	116.2 ± 22.8	129.5 ± 20.3	−4.21	<0.01
	3–4 (*n* = 57)	125.8 ± 24.6	138.2 ± 21.5	−2.18	0.03
Blood loss (mL)	0–2 (*n* = 140)	38.4 ± 9.1	52.6 ± 10.2	−5.63	<0.01
	3–4 (*n* = 57)	48.6 ± 11.3	56.2 ± 12.8	−1.76	0.08
Tumor resection rate (%)	0–2 (*n* = 140)	88.2 ± 6.4	87.5 ± 6.9	0.72	0.47
	3–4 (*n* = 57)	85.1 ± 7.8	84.3 ± 8.2	0.41	0.68

Data presented as mean ± standard deviation. Knosp grade 0–2 indicates limited parasellar extension; grade 3–4 indicates cavernous sinus invasion.

### 3.3 Comparison of complications between gauze packing and non-gauze packing

Among patients with gauze packing in both nasal cavities after surgery, 6 had diabetes insipidus, and 1 had cerebrospinal fluid rhinorrhea. In contrast, among patients without gauze packing, 57 had diabetes insipidus, and 26 had cerebrospinal fluid rhinorrhea see Table 5. The differences were statistically significant. There were no statistically significant differences in intracranial infection, visual field damage, follicle-stimulating hormone, luteinizing hormone, growth hormone, and olfactory tests between the two groups. However, given the retrospective design and absence of standardized packing criteria, these associations should be interpreted cautiously and do not establish causality.

**Table 5:** Comparison of complications between gauze packing and Non-gauze packing.

Item	Gauze Packing (*n* = 39)	Non-Gauze Packing (*n* = 158)	χ^2^ value	*P* value
Diabetes Insipidus	6 (15.4%)	57 (36.1%)	5.13	0.02
Cerebrospinal Fluid Rhinorrhea	1 (2.6%)	26 (16.5%)	3.84	0.05
Intracranial Infection	0 (0%)	6 (3.8%)	0.72	0.47
Visual Field Damage	0 (0%)	1 (0.6%)	—	1.00
Olfactory Test (T&T Score)				
3 Days Postoperatively	1.20 (0.60–1.70)	1.20 (0.60–1.50)	0.64	0.52
1 Week Postoperatively	2.10 (1.50–2.55)	2.00 (1.60–2.60)	0.30	0.76
1 Month Postoperatively	3.10 (2.55–3.50)	3.30 (2.70–3.90)	1.48	0.14

Data are presented as *n* (%) or median (interquartile range). Categorical variables are compared using χ^2^ test or Fisher's exact test. Continuous variables are compared using Mann–Whitney U test.

### 3.4 Cost-effectiveness analysis and long-term economic burden by surgical approach

Cost-effectiveness analysis revealed the transseptal approach to be economically dominant (ICER: 255.12 vs. 287.32 CNY per unit effectiveness). Long-term economic burden was comparable between approaches, with no significant differences in re-operation, recurrence, or hormone replacement therapy costs (Table 6).

**Table 6:** Cost-effectiveness analysis and long-term economic burden by surgical approach.

Item	Transseptal Approach (*n* = 108)	Bilateral Nostril Expanded Approach (*n* = 89)	T/Z/χ^2^ value	*P* value
Cost-effectiveness				
Incremental Cost-Effectiveness Ratio (CNY per unit composite effectiveness)*	255.12	287.32	—	—
Long-term Economic Burden				
Re-operation Cases	8 (7.4%)	7 (7.9%)	0.017	0.90
Recurrence Cases	16 (14.8%)	14 (15.7%)	0.05	0.82
Hormone Replacement Therapy Cost (CNY)	799 (772–836)	812 (777–834)	1.34	0.18

*Data are presented as mean ± standard deviation, median (interquartile range), or *n* (%). Cost-effectiveness analysis uses “operation time shortened by 11 min/intraoperative blood loss reduced by 20 mL” as the effectiveness indicator to calculate the incremental cost-effectiveness ratio (ICER).

## 4 Discussion

Endoscopic endonasal surgery has gradually supplanted microscopic techniques to become the acknowledged minimally invasive approach among neurosurgeons, owing to its multiple advantages (8). Although the endoscopic transnasal technique is well established, selecting the optimal corridor and exploring novel routes—without compromising the extent of resection—remain active areas of investigation (9). Consequently, a substantial body of literature has focused on the impact of different surgical corridors on postoperative morbidity (10–13). The trans-septal approach and the bilateral-nostril extended transsphenoidal approach described in these reports are both bilateral transnasal routes. To date, no study has specifically examined the relationship between bilateral transnasal approaches and outcomes in pituitary adenoma surgery. In the present study, neither the trans-septal nor the bilateral-nostril extended transsphenoidal approach demonstrated significant differences in medical costs, extent of resection, or complication rates; furthermore, our data indicate that the trans-septal approach is superior to the bilateral-nostril extended transsphenoidal approach in reducing operative time and intraoperative blood loss.

In the present cohort, the trans-septal approach reduced mean operative time by 11 min and intra-operative blood loss by 20 mL compared with the bilateral-nostril extended approach. To mitigate selection bias, we performed variable ratio propensity-score matching (PSM) with a caliper of 0.02, adjusting for age, sex, tumor size, and Knosp grade. This approach was chosen to optimize statistical power given the limited sample size in the bilateral approach group, resulting in 108 transseptal patients matched to 89 bilateral approach patients. After matching, covariate balance was satisfactory (all standardized mean differences <0.1), and the difference in the primary outcome remained consistent with that observed in the full cohort. Three anatomical and technical factors account for this advantage.(1) Shorter anatomical trajectory: the distance from the anterior nasal spine to the anterior wall of the sphenoid sinus measures 5.2–5.8 cm via the trans-septal corridor, whereas the bilateral extended route requires additional dissection of the bilateral nasal vestibular mucosa and fracturing of the bony septum, lengthening the working channel by 0.7–1.0 cm.(2) Preservation of mucosal flap vascularity (14): the trans-septal technique elevates only a unilateral mucoperichondrial–mucoperiosteal flap, leaving the contralateral branches of the sphenopalatine artery intact. Comparison of hormone levels with and without gauze packing, see Table 7. This minimizes pulsatile bleeding caused by bilateral mucosal lacerations (3). Reduced bony fenestration (15): exposure of the sellar floor is achieved through a 1.2–1.5 cm^2^ anterior sphenoidotomy in the trans-septal group, whereas the bilateral extended approach necessitates bilateral sphenoidotomies and partial resection of the sphenoid crest, enlarging the bony window by 30%–40% and increasing cancellous bone oozing. Our subgroup analyses by Knosp grade provide important guidance for surgical planning. The transseptal approach demonstrated clear advantages in operative time and blood loss for Knosp grade 0−2 tumors, supporting its preferential use for these cases. However, for Knosp grade 3–4 tumors with extensive cavernous sinus invasion, the visual axis and instrument maneuverability afforded by the transseptal corridor may become insufficient. In our cohort, 13 patients (12.0%) in the transseptal group and 44 (49.4%) in the bilateral group had Knosp grade 3–4 tumors. The demonstrated reductions in operative time and blood loss were restricted to pituitary adenomas classified as Knosp grade 0–2 with predominantly intrasellar or minimal suprasellar extension. When the neoplasm extensively invades the lateral cavernous sinus compartment, protrudes into the third ventricle, or displays broad parasellar extension, the visual axis and instrument maneuverability afforded by the trans-septal corridor may become insufficient (16). Consequently, preoperative evaluation must integrate MRI-based Knosp grading, Hardy classification, and surgeon experience; conversion to an extended endoscopic approach (EEA) or a staged transcranial–endonasal strategy should be considered whenever necessary to ensure safe oncological resection and preservation of neurovascular structures.

**Table 7:** Comparison of hormone levels with and without gauze packing.

Intra-op gauze use	Pre-operative hormone abnormalities (%)				Post-operative hormone abnormalities (%)			
	Prolactin	TSH	FSH	GH	Prolactin	TSH	FSH	GH
Gauze packing (*n* = 39)	23 (59%)	3 (7.7%)	20 (51.3%)	34 (87.2%)	13 (33.3%)	13 (33.3%)	3 (7.7%)	3 (7.7%)
No gauze packing (*n* = 158)	106 (67.1%)	6 (3.8%)	67 (42.4%)	50 (31.6%)	77 (48.7%)	7 (4.4%)	19 (12%)	5 (3.2%)
*Χ*2 value	1.94	1.03	0.022	9.79	1.23	11.51	0.61	0.647
*P* value	0.16	0.31	0.88	<0.01	0.27	<0.01	0.43	0.42

Data are presented as *n* (%). TSH: thyroid-stimulating hormone; FSH, follicle-stimulating hormone; GH, growth hormone.

Postoperative nasal packing remains controversial. In this retrospective cohort, packing was only statistically associated with complications; causality cannot be inferred because of potential confounders (e.g., sellar defect size, CSF leak grade). Packing stabilizes reconstruction and dampens CSF pressure fluctuations (7), yet we observed a transient TSH rise. Probable mechanisms include pituitary-stalk traction, local inflammation (IL-6, TNF-α), and mild hypoxia from increased airway resistance. We contend that TSH elevation attributable to packing is transient and self-limiting, with negligible long-term impact on endocrine function or quality of life; routine intervention is therefore unnecessary. However, we recommend routine reassessment of pituitary–target gland hormones at 4–6 weeks postoperatively. Persistent abnormalities should prompt collaborative management with endocrinology to avert overlooked hypopituitarism. In clinical practice, the decision to employ nasal packing should be individualized, weighing its benefits against potential drawbacks while considering tumour extent, adequacy of sellar repair, and patient-specific factors.

Current expert consensus and guidelines (17, 18) emphasize comprehensive disease management but devote limited attention to patient-level economic burden. Even in high-volume centers, 10%–30% of patients harbor imaging-documented residual or early recurrent pituitary adenoma. Stereotactic radiosurgery—Gamma Knife or CyberKnife—can achieve 5-year tumor control rates of 85%–95% and hormonal remission rates of 50%–70% while obviating reoperation (19). Our data show no difference in reoperation (7.4% vs. 7.9%) or recurrence (14.8% vs. 15.7%) between the trans-septal and bilateral-nostril extended approaches, underscoring the comparable need for adjuvant radiosurgery in both cohorts. Multidisciplinary teams should therefore identify candidates for stereotactic treatment within 3–6 months via MRI and endocrine evaluation, minimizing long-term medical and psychological costs. A cost-effectiveness analysis was performed with “11-min reduction in operative time and 20-mL decrease in blood loss” as effectiveness endpoints. The incremental cost-effectiveness ratio (ICER) demonstrated that the trans-septal approach was economically dominant over the bilateral-nostril extended transsphenoidal approach. Although the 20-mL blood-sparing difference *per se* may be clinically modest, it serves as a quantifiable proxy for reduced surgical trauma and faster recovery, which together with the 11-min shorter operative time, rendered the transseptal approach dominant in our model. We therefore regard the bleeding difference as a “signal of benefit” rather than a standalone clinical endpoint.

Using a purpose-designed economic-burden questionnaire (20), we documented substantial financial strain in the majority of households. High out-of-pocket costs frequently led to deferred therapy or follow-up, compromising overall outcomes. HADS scores indicated mild anxiety or depression in nearly 40% of patients, driven by disease uncertainty, operative risk, and post-operative quality-of-life concerns. Importantly, these psychological disturbances did not differ significantly between surgical approaches, indicating they reflect the overall burden of pituitary adenoma diagnosis and treatment rather than the specific technical approach. Recent neuroimaging evidence suggests that these symptoms may also have an organic basis in pituitary neuroendocrine tumors. Specifically, Gökoğlu et al. (21) demonstrated significant volumetric reductions in the limbic system and hippocampus in PitNET patients, correlating these structural alterations with emotional impairments. This neuroanatomical perspective complements our observation that psychological burden is related to the disease and surgical experience overall rather than the specific nasal corridor selected.hese psychological disturbances not only impair health-related quality of life but may also impede immune function and convalescence through neuroendocrine pathways, creating a vicious cycle.

Optimal management of pituitary adenomas demands seamless collaboration among neurosurgery, endocrinology, ophthalmology, radiology, psychology, and other disciplines; multidisciplinary team (MDT) care has therefore become pivotal for improving outcomes (22). The “Pituitary Center of Excellence” model adopted by leading institutions worldwide integrates subspecialty expertise to deliver patient-centered, end-to-end care, thereby enhancing diagnostic accuracy and therapeutic success (23). Our findings underscore the urgent need to strengthen MDT collaboration in pituitary disease management. Going forward, centers should establish formal pituitary MDTs with clearly defined roles and standardized workflows, dismantling traditional silos to enable information sharing and resource integration. Concurrently, telemedicine and artificial intelligence should be leveraged to broaden the reach and depth of MDT collaboration, improving access to high-quality, standardized, and precision care for all patients with pituitary adenomas.

As a single-center retrospective study, our findings are subject to selection bias, regional homogeneity, and missing data. We therefore plan a multicenter, prospective, randomized controlled trial with standardized data collection and extended follow-up to assess long-term quality of life, endocrine recovery, and tumor recurrence, thereby enhancing the level of evidence and generalizability.

## 5 Conclusion

This single-center retrospective comparison demonstrated that the trans-septal approach reduced operative time and intra-operative blood loss relative to the bilateral-nostril extended transsphenoidal route, whereas the two corridors were equivalent with respect to medical costs, psychological burden, endocrine profiles, and peri-operative complications. Postoperative nasal packing was associated with fewer complications, but this observation awaits confirmation in large, multicentric studies. The substantial economic and psychological loads borne by patients with pituitary adenomas mandate intensified multidisciplinary collaboration and optimized pharmacotherapy to lower overall costs and improve long-term outcomes.

## Data Availability

The raw data supporting the conclusions of this article will be made available by the authors, without undue reservation.
